# Perceptual Adaptation to the Correction of Natural Astigmatism

**DOI:** 10.1371/journal.pone.0046361

**Published:** 2012-09-26

**Authors:** Maria Vinas, Lucie Sawides, Pablo de Gracia, Susana Marcos

**Affiliations:** Instituto de Óptica, Consejo Superior de Investigaciones Científicas (CSIC), Madrid, Spain; Dalhousie University, Canada

## Abstract

**Background:**

The visual system adjusts to changes in the environment, as well as to changes within the observer, adapting continuously to maintain a match between visual coding and visual environment. We evaluated whether the perception of oriented blur is biased by the native astigmatism, and studied the time course of the after-effects following spectacle correction of astigmatism in habitually non-corrected astigmats.

**Methods and Findings:**

We tested potential shifts of the perceptual judgments of blur orientation in 21 subjects. The psychophysical test consisted on a single interval orientation identification task in order to measure the perceived isotropic point (astigmatism level for which the image did not appear oriented to the subject) from images artificially blurred with constant blur strength (B = 1.5 D), while modifying the orientation of the blur according to the axis of natural astigmatism of the subjects. Measurements were performed after neutral (gray field) adaptation on naked eyes under full correction of low and high order aberrations. Longitudinal measurements (up to 6 months) were performed in three groups of subjects: non-astigmats and corrected and uncorrected astigmats. Uncorrected astigmats were provided with proper astigmatic correction immediately after the first session. Non-astigmats did not show significant bias in their perceived neutral point, while in astigmatic subjects the perceived neutral point was significantly biased, typically towards their axis of natural astigmatism. Previously uncorrected astigmats shifted significantly their perceived neutral point towards more isotropic images shortly (2 hours) after astigmatic correction wear, and, once stabilized, remained constant after 6 months. The shift of the perceived neutral point after correction of astigmatism was highly correlated with the amount of natural astigmatism.

**Conclusions:**

Non-corrected astigmats appear to be naturally adapted to their astigmatism, and astigmatic correction significantly changes their perception of their neutral point, even after a brief period of adaptation.

## Introduction

It is well known that the visual coding is a dynamic process, adapting continuously to changes in the visual context (for instance, changes in the contrast, luminance, blur or color in the visual scene), or changes in the observer him/herself (for example by disease, treatment, aging, or a new spectacle prescription) [1]. Adaptation is therefore related to the adjustment of the visual system to changes in the environment, as well as to recalibrations to changes within the observer, which allow maintaining a match between visual coding and visual environment throughout the life span. However, the time-scale for these adaptation processes has only been marginally investigated. Most studies measure adaptation as changes in sensitivity over milliseconds to minutes [2], [3]. However, it is likely that these adjustments operate for hours, weeks or even years.

An interesting debate is the relationship between adaptation and perceptual learning [1]. Adaptation is typically characterized by an immediate shift in perceptual appearance of a scene after a typically brief exposure to a modified visual experience. On the other hand, perceptual learning is normally characterized by a longer time course [4], leading to changes not only in visual appearance, but also on visual performance [5]. However, the line between adaptation and perceptual learning is blurred by the fact that learning can actually produce changes in the appearance of visual scenes [6], and some adaptation processes can actually operate at long time-scales, can show persistent after-effects, and in fact exhibit some forms of learning [7].

Among the most straightforward ways to modify the appearance of the visual world to investigate the processes underlying visual adaptation is image blurring. This can be achieved by lenses, or by computer simulations of image blurring/sharpening. The advent of Adaptive Optics has actually allowed modifying the high order aberrations of the eye, therefore allowing high control over the amount and specific form of the blur of the retinal image of the subjects [8], [9], [10]. Following brief periods of adaptation to blurred or sharpened images, subjects show shifts in the perceived blur [11]. Recent studies have shown that subjects can adapt to the blur produced by defocus as well as high order aberrations (scaled versions of their own aberrations, or other subjects' aberrations) [12], [13]. These short-term after-effects appeared in both perceived blur and visual acuity, following exposure to blur introduced optically or by filtering images [11], [14], [15]. There is also evidence that with longer exposures to blur (by lenses, surgically-induced, or resulting from a corneal condition) [14], [16], [17], [18] adaptation may lead to improvements in visual acuity, perhaps by some form of perceptual learning. Interestingly, there is increasing evidence that observers appear to be adapted to the blur level produced by their high order aberrations, as the level of blur that produces no after-effects matches the native blur level in subjects [12], [13], [19]. However, the role of orientation of the specific form of blur remains to be elucidated.

Astigmatism is a low order aberration, but the inherent oriented nature of the blur that it produces makes it particularly attractive to investigate adaptive processes in the visual system. Astigmatism occurs in 85% of the population [20], and can be easily corrected (or induced) by cylindrical lenses. Uncorrected astigmatism in adults causes significantly decreased visual performance [20], [21]. Also, numerous studies have shown that large amounts of astigmatism left uncorrected in childhood may lead to meridional visual deficits, so called meridional amblyopia, although those are not found in all visual tasks [22], [23].

Adaptation to astigmatism, and in particular, to a newly prescribed correction of astigmatism, is particularly relevant clinically, where the optometrist or the surgeon faces the decision of astigmatism correction by spectacles, contact lenses, intraocular lenses or corneal surgery. In a recent study we showed strong after-effects after brief periods of adaptation to images blurred with astigmatism (while keeping the blur strength constant), indicating that adaptation can be selective to the orientation of astigmatism [24]. Ohlendorf et al. reported an increase of visual acuity in normal subjects viewing dynamic astigmatic images (either simulated, or through +3 D cylindrical lenses) after 10 min of adaptation, with a significant meridional bias [25].

Potential common mechanisms underlying adaptation and perceptual learning have also been explored using induced astigmatism as a probe. Yehezkel et al. (2010) pointed that the process of adaptation to astigmatic lenses (2 and 4 hrs) might exhibit forms of learning. The course of this adaptation, presence of after-effects, and accumulative effects over sessions (consistent with perceptual learning) differed in two groups of subjects, treated monocularly with the contralateral eye covered or uncovered (dichoptic group), indicating a binocular cortical site of adaptation [7].

The previous studies investigated the pattern of adaptation to astigmatism in non-astigmatic eyes. In the current study we will investigate the adaptation process to an astigmatic correction in astigmatic subjects. A previous study suggested that habitually non-corrected astigmats were adapted to their astigmatism, as their measured visual acuity was less impaired by the induction of astigmatism than in non-astigmatic subjects with the same amount of induced astigmatism [26]. This may be also the result of a form of perceptual learning. A similar finding has been described in keratoconic patients (with highly optically degraded corneas), who showed a better performance than normal subjects with simulated identically degraded optics [18], [27]. However, to our knowledge the course of neural adaptation to an astigmatic correction has not been investigated.

In this study, adaptation will be specifically tested by measuring the astigmatic stimulus level which appears neutral (non-oriented) in corrected-astigmats, and in the latter-corrected astigmats, and subsequently, after an astigmatic prescription was given to the astigmats (2 hours to 6 months). We expect that the level of astigmatism that appears neutral (non-oriented) to the subjects corresponds to a perceptual norm, which reflects a balance in the underlying neural response. To what extent this perceptual norm changes after adaptation to a refractive correction, and the time course for this adaptation has not been, to our knowledge, investigated before. Alternatively, these experiments will allow exploring whether there may be learned properties in astigmats, which may persist despite the presence of an adapting stimulus. A previous study [7] actually pointed out to the learned ability of storing multiple transformations of the visual world, allowing observers to switch between two different optical corrections that induced different visual distortions.

## Materials and Methods

The experiment was designed to test potential shifts of the perceived neutral point of the astigmatic subjects before and after adaptation to a new astigmatic spectacle correction in comparison with non-astigmats and habitually-corrected astigmats, by using a psychophysical test. A series of artificially blurred images, with constant blur strength, but orientation tuned to the axis of natural astigmatism of the subjects, were used to estimate deviations from the isotropically blurred image.

### Ethics Statement

All participants, who were acquainted with the nature of the study, provided written informed consent. All protocols met the tenets of the Declaration of Helsinki and had been previously approved by the Consejo Superior de Investigaciones Científicas (CSIC) Ethical Committee.

### Subjects

The sample consisted of 21 subjects (ages ranging from 23 to 51 years (31.77±7.99)). Subjects were selected *a priori*, and classified according to their natural astigmatism and whether this was habitually corrected or not. All subjects followed an exhaustive optometric evaluation at the University Complutense de Madrid School of Optometry Clinic.

The subjects were classified in three groups (n = 7 per group): G1 (control group of subjects with no clinical astigmatism); G2 (astigmatic subjects, habitually corrected, wearing an astigmatic correction since childhood); G3 (astigmatic subjects, habitually-non-corrected). The inclusion criterion for G1 was that astigmatism was lower than 0.25 D. Inclusion criteria for G2 and G3 were: (1) natural astigmatism ≥0.75 D; (2) Myopic astigmatism. Tests were performed only on one eye per subject (less myopic eye in G1; and less myopic eye with ≥0.75 D of astigmatism in G2 and G3).

All subjects in G3 were provided with astigmatic spectacle correction of their natural astigmatism after an initial test. Table 1 shows the profile and refraction state of all subjects of the study (the measured eye indicated in bold). For G2, spherical error ranged from −5.25 to 0.25 D (mean −2.56±1.87 D), while for G3 spherical error ranged from −1.50 to 0.25 D (mean −0.39±0.64 D). Refractive errors were measured using standard clinical optometric procedures.

**Table 1:** Subjects' profile.

	OD			OS			Blur	Age
ID	Sph	Cyl	Axis	Sph	Cyl	Axis	axis	
**G1_A**	**0.50**	**–**	**–**	0.50	–	–	–	29
**G1_B**	**0.00**	**–**	**–**	0.00	–	–	–	33
**G1_C**	**0.00**	**–**	**–**	0.00	–	–	–	31
**G1_D**	**0.00**	**–**	**–**	0.00	–	–	–	30
**G1_E**	**−0.25**	**−0.25**	**80**	−0.25	–	–	170	30
**G1_F**	**0.25**	**−0.25**	**90**	0.25	−0.25	80	90	34
**G1_G**	**0.00**	**–**	**–**	0.00	–	–	–	23
**G2_A**	**−3.50**	**−1.00**	**10°**	−4.00	−1.25	170°	100	33
**G2_B**	**−5.25**	**−1.25**	**105°**	−6.00	−1.50	90°	15	27
**G2_C**	**−4.00**	**−1.00**	**75°**	−3.75	−0.50	115°	165	34
**G2_D**	**−0.75**	**−1.25**	**90°**	−1.25	−0.75	85°	0	30
**G2_E**	**−2.25**	**−0.75**	**90°**	−2.00	−0.75	90°	0	51
**G2_F**	−4.50	−0.50	30°	**−1.75**	**−1.00**	**170°**	80	31
**G2_G**	0.25	−1.00	175°	**0.25**	**−1.25**	**175°**	85	23
**G3_A**	**−1.50**	**−0.75**	**10°**	−1.50	−0.75	155°	100	27
**G3_B**	0.25	−1.75	95°	**0.00**	**−1.25**	**80°**	170	29
**G3_C**	**−0.75**	**−0.75**	**120°**	−1.00	−0.50	40°	30	27
**G3_D**	**0.50**	**−0.75**	**170°**	2.00	−5.00	175°	170	27
**G3_E**	−0.75	−0.75	130°	**−0.75**	**−0.75**	**175°**	85	48
**G3_F**	−1.25	−0.50	90°	**−1.00**	**−0.75**	**90°**	0	45
**G3_G**	**0.00**	**−1.00**	**90°**	0.25	−1.00	75°	0	26

Optometric subjective refractions (spherical error, cylinder, axis), orientation of the retinal blur, and ages. The measured eye is shown in bold.

### Adaptive optics system

A custom-made Adaptive Optics system was used to characterize and correct the aberrations of the subject, therefore controlling the blur of the images projected on the retina. The set-up has been described in detail in previous publications [10], [28], [29]. In brief, the main components of the system are a Hartmann-Shack wavefront sensor (32×32 microlenses, 3.6 mm effective diameter; HASO 32 OEM, Imagine Eyes, France), a Super Luminescent Diode (827 nm for illumination), an electromagnetic deformable mirror (52 actuators, a 15-mm effective diameter and a 50 µm stroke; MIRAO, Imagine Eyes, France), a motorized Badal system, a natural pupil monitoring system, and a stimulus display. All optoelectronic components of the system were controlled with custom software in C++. The state of the mirror that compensates the aberrations of the subject was found in a closed-loop operation, and continuous measurements of the subjects' aberrations throughout the test ensured proper correction. Measurements were performed for 6-mm pupils (limited by an artificial pupil), under natural viewing conditions.

Visual stimuli were presented on a CRT monitor (Mitsubishi Diamond Pro 2070) through the Badal and AO mirror correction. The stimulus display was controlled by the psychophysical platform ViSaGe, (Cambridge Research System, UK). The average luminance (after losses in the system) was around ∼30 cd/m^2^ in an otherwise dark environment.

### Generation of the test images

A Perlin noise image was used as a stimulus test (480×480 pixels, 1.98 deg angular subtend). Perlin noise is a procedural texture based on lattice gradient noise [30], which is easily modulated using two computational parameters, the base frequency and persistence [31]. This type of noise produces a repeatable pseudo-random value for each input position, has a known range and band-limited spatial frequency, does not show obvious repeating patterns, and its spatial frequency is invariant under translation [32], which makes it especially suitable for studying astigmatic images. The Perlin noise image was generated with a Perlin Noise Generator Software [33], with the following inputs: persistence 0.7; octaves 8; zoom 16; random seed; normalized noise. The root-mean-square (RMS) contrast of the stimuli was 0.69 calculated following Peli et. al (1990) [34]. Images were blurred using custom algorithms to simulate optical blur by convolving the images with the point spread functions (PSF) corresponding to different levels of astigmatism and defocus, but constant blur strength.

Blur strength (B) is typically defined as 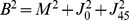 (1), where M, J_0_, and J_45_, in diopters, represent equivalent defocus, vertical/horizontal astigmatism, and oblique astigmatism, respectively [35]. Equation (1) expressed in microns is as follows: 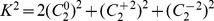 (2), where 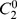, 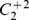 and 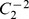 are the Zernike terms for defocus, vertical and oblique astigmatism respectively, and 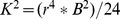 (3) (with *r* = pupil radius in meters). Each combination of astigmatism and defocus produced the same amount of blur strength (*B* = 1.5 D in diopters, or *K* = 2.75 µm in microns). For example, to generate a set of images varying from astigmatism at 0 to 90°, 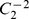 was set to 0, and 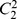 was varied from −2 to +2 µm in 0.02 µm steps (or equivalently from −1.09 to 1.09 D in 0.01 D steps). Simultaneously, the defocus term was varied to keep the blur strength constant between 1.34 µm (or −1.03 D) when astigmatism was ±2 µm, and 1.95 µm (or −1.5 D) when astigmatism was set to 0, using similar procedures to those described by Sawides et al. 2010 [24].

In G1 the axis of astigmatic blur varied from 0° to 90° (vertically to horizontally oriented astigmatic blur). In G2 and G3 the axis of astigmatic blur was matched to the subject's axis of natural astigmatism, varying the orientation of astigmatic blur following the natural axis of astigmatism to a 90° rotated axis.

**Figure 1:**
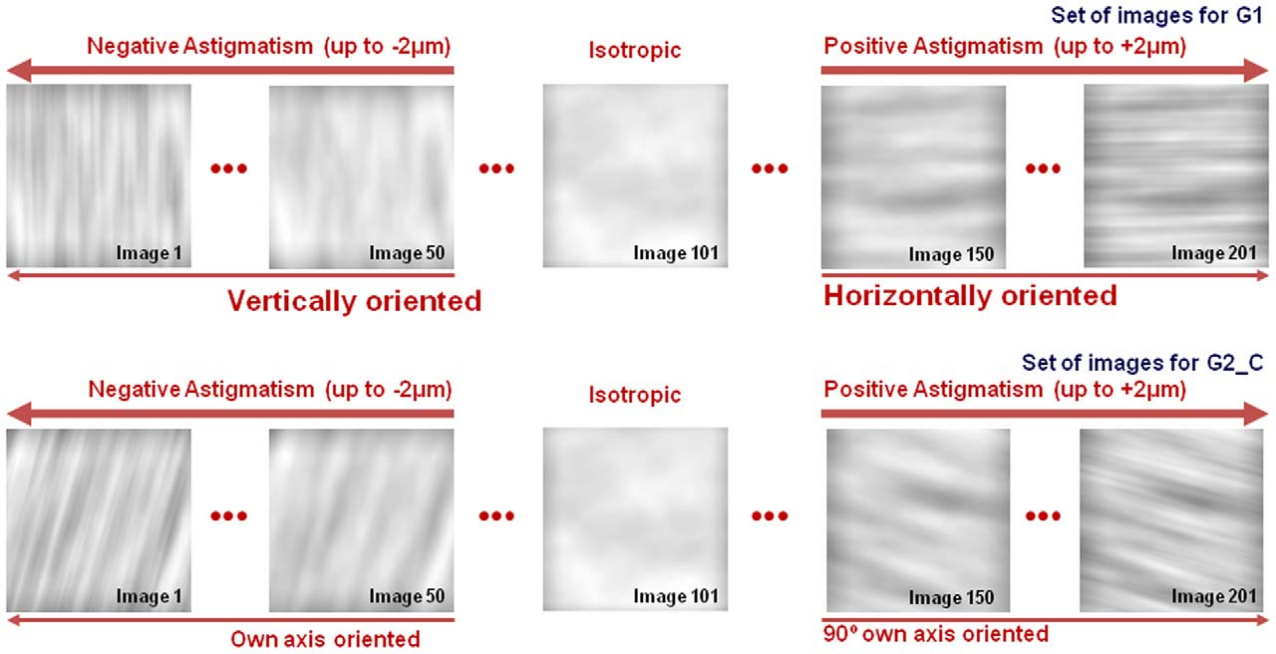
Examples of test images series. Astigmatic blur was generated by varying astigmatism (from −2 µm to +2 µm), and defocus to maintain constant blur strength (B = 1.5 D). Image 101 was isotropically blurred. Top panel: image series presented to all subjects from G1, with vertically oriented blur (images 1–100) to horizontally oriented blur (images 102 to 201). Bottom panel: an example of image series presented to G2/G3 (the example corresponds to G2_C in particular, with images blurred along the axis of natural astigmatism of the eye (75°) to a 90° rotated axis (165°).

A total of 201 images were generated for each test, with constant blur and varying relative contribution of defocus and astigmatism (ranging from negative to positive). Figure 1 shows a typical example of sets of test images for G1 subjects (upper panel) and for one of the astigmatic subjects (lower panel), G2_C (Astigmatism: −1.00×75°).

### Experimental protocol and psychophysical paradigm

Measurements were performed monocularly, under natural viewing conditions and naked eyes in a darkened room. Measurements were performed always in the same eye of the subject. The eye's pupil was aligned to the optical axis of the instrument, and the subject's was stabilized using a dental impression. Astigmatism and high order aberrations were measured and corrected in a closed loop adaptive optics operation. The subject was then asked to adjust the Badal system position to achieve best subjective focus. The state of the mirror that achieved the correction was saved and applied during the measurements. Psychophysical measurements were performed under full static AO-corrected aberrations and best spherical refraction error correction.

Subjects performed a single stimulus detection task, in which the observer sets his/her own internal criteria for response (in this case, their perceived neutral point) [36]. The psychophysical paradigm consisted of a single interval orientation identification task [37], [38], used to detect the threshold for astigmatism orientation, while using a QUEST (Quick Estimation by Sequential Testing) algorithm (maximum likelihood estimator, from the Psychtoolbox package) [39] to calculate the sequence of presented stimulus (level of astigmatic blur in the image) in the test, following the subject's response. The subject had to report the perceived orientation (between two different axes) from a series of images in order to estimate the threshold/perceived isotropic point (the image that appears non-oriented to the observer). The QUEST routine usually converged after less than 40 trials, where the threshold criterion was set to 75%. The threshold was estimated as the average of the 10 last stimulus values, which oscillated around the threshold with standard deviation below 0.03 µm.

Before the measurement, subjects were instructed on the required responses according to their perceived image orientation. Non-astigmatic subjects and astigmatic subjects with natural astigmatism, which were presented with images oriented vertically or horizontally had to respond up or down respectively. Astigmatic subjects with astigmatism different form 0° or 90° were presented with images oriented at the axis of their natural astigmatism or at the perpendicular axis, and had to respond right or left respectively. Subjects used a response box from Cambridge Research Systems. The experiments were performed after the subject adapted for 5 s to gray field, and then the test images were presented for 1.5 s. The gray field was presented again between images, for 1 s, during which the subject had to respond.

Subjects performed the same astigmatic blur judgments in 4 different sessions for G1 and G2: first session (S0A), and after 1 week (S1), 1 month (S2), and 6 months (S3). G3 subjects were prescribed with spectacle refractive correction, which compensated their uncorrected astigmatism. Measurements in G3 were performed in a first session, before correction wear (S0A), on the same first day, after 2-hours of correction wear (S0B), and after 1 week (S1), 1 month (S2), and 6 months (S3) of astigmatic correction wear. Subjects G3_A and G3_B did not perform session S1. In each session, the test was repeated 4 times for each subject.

### Control experiment: the oblique effect

It is well known that, even in the absence of astigmatism, oblique gratings are less visible than gratings oriented at 0/90° [40], and that orientation sensitivity is lower at oblique axes that at the cardinal axes [41]. Unlike for visual performance tasks [42], [43], previous studies have shown that both oblique and 0/90 targets are equally effective in an adaptation experiment [44]. Nevertheless, we conducted a control experiment to ensure that results on non-astigmatic subjects (where the adaptation test was performed using targets blurred along the cardinal axes) were not affected by the selected orientation. The experiment was performed on two non-astigmatic subjects (G0_A and G0_B; ages: 26 and 32; spherical error ≤0.25 D), with astigmatic blur imposed at 0/90° and at 45/135°.

### Analysis of results

The perceived isotropic image (which did not appear oriented to the subject) was measured for each subject and session, and the corresponding astigmatic blur and axis were estimated. Data were obtained from the image chosen as isotropic by each subject (4 repeated times per trial), converted into amount of astigmatism, and averaged to obtain the average perceived neutral point (in terms of microns of astigmatism) for each measurement session. Shifts of the isotropic point from the first session (S0A) were analyzed to test potential longitudinal variations of the perceived isotropic point (and after correction of astigmatism in G3). Also, the total shift of the isotropic point (from S0A to S3) was analyzed as a function of the amount of natural astigmatism of the subjects. Statistical analysis with SPSS software (IBM SPSS Statistic Software) was performed to test differences in perception of neutral point across sessions (paired-samples t-test), and also to test the relationship between natural astigmatism and longitudinal variations, as well as the differences between groups and variables of the study (one-way ANOVA).

## Results

Adaptation to astigmatism was measured with a series of psychophysical tests (under full-adaptive optics correction) in order to measure the perceived isotropic point (astigmatism level for which the image did not appear oriented to the subject) from images artificially blurred with constant blur strength but orientation tuned to the axis of natural astigmatism of the subjects.

### Subjects' natural aberrations

**Figure 2:**
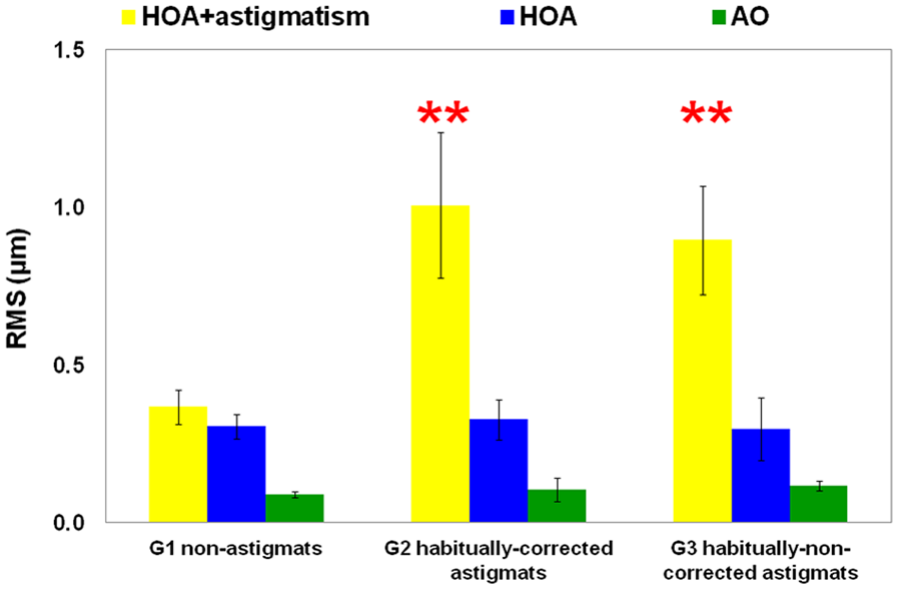
RMS wavefront error. RMS wavefront error for natural aberrations for HOAs and astigmatism (yellow bars), HOAs (blue bars), and, residual aberrations after AO correction (green bars), averaged across subjects, groups and measurement sessions. Error bars indicate inter-subject variability. ** indicates a significantly larger RMS (p<0.01) for HOA+astigmatism for G2 and G3 than for G1.

Figure 2 shows the average ocular Root-Mean Square wavefront error (RMS) for high order aberrations (HOA) (RMS_HOA_, blue bars), for HOA and natural astigmatism (RMS_HOA+ast_, yellow bars), and for residual aberrations after AO-correction of all natural aberrations (RMS_AO_, green bars), in each group. As expected, RMS_HOA+ast_ was significantly higher for G2 and G3 than for G1 (one-way ANOVA; F(2,18) = 6.881, p = 0.006). RMS_HOA_ was similar across the 3 groups (one-way ANOVA; F(2,18) = 0.403, p = 0.674), although the contribution of HOA to the RMS_HOA+ast_ differs across groups: HOA contributes on average with 85% in G1, 36% in G2, and 33% in G3. The experiments were performed under correction of both HOA and astigmatism. RMS_AO_ was similar across the different groups.

### Shifts of the perceived neutral point

Potential changes in the perception of the neutral point of the subjects were studied. In the control experiment in two non-astigmatic subjects, the shift in the perceived neutral point for the two tested orientations was similar (G0_A: 0.13 µm for 0/90° and 0.14 µm for 45/135°; G0_B: 0.11 µm for 0/90° and 0.11 µm for 45/135°). Also, the perceived neutral point was not statistically significantly different from the isotropic point (paired samples t-test; t (3) = 0.002; p>0.6).

**Figure 3:**
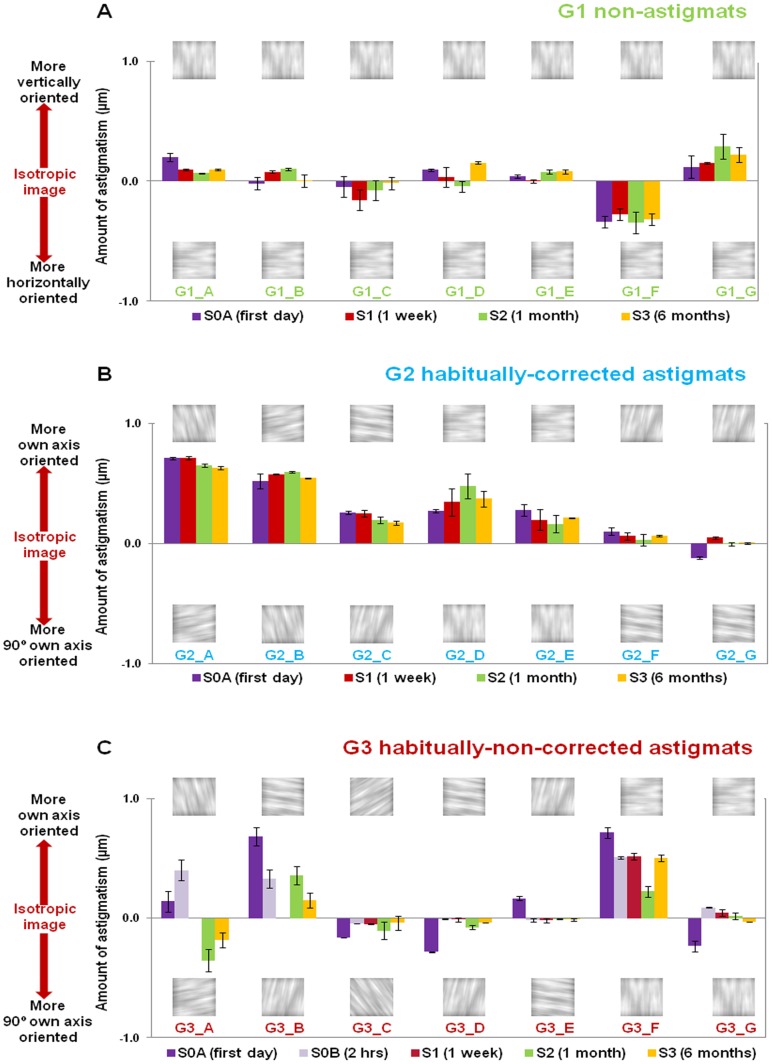
Perceived neutral point. Perceived neutral point (µm of astigmatism) for all subjects and in all sessions (First session: purple bars; 2-hrs: violet bars; 1-week: red bars; 1-month: green bars; 6-months: yellow bars). A: G1, non-astigmats. B: G2, habitually-corrected astigmats. C: G3, habitually-non-corrected astigmats. For illustration purposes, the first and last images of the series are shown (−2 µm to +2 µm of astigmatism). G1: 0°/90°; G2 and G3: tuned to the axis of natural astigmatism/90°. Error bars stand for intra-subject variability (standard deviation) for repeated measurements (4 times/test).

Figure 3 shows the average deviations from the isotropic point (in terms of amount of astigmatism), obtained from the image chosen as isotropic by each subject (data averaged from the 4 repeated measurements in each session) in the QUEST procedure. For representation purposes we refer to positive perceived neutral point to that oriented vertically (G1) or to the axis of natural astigmatism (G2 and G3). Also, negative perceived neutral point is that oriented horizontally (G1) or that oriented perpendicularly to the axis of natural astigmatism (G2 and G3). The error bars represent the standard deviation for 4 repeated measurements in each subject. Non-astigmats (G1, Figure 3-A) judged as isotropic images predominantly blurred by symmetric blur. On average, deviation from the isotropic point (absolute values) at S0A for G1 was 0.12 µm. Only G1_F and G1_G showed some bias towards horizontal and vertical astigmatism, respectively. Also, the perceived neutral point remained constant across sessions for all subjects in G1.

Most of the habitually corrected astigmats (G2, Fig. 3-B) showed a bias in the perceived neutral point towards their axis of natural astigmatism (Figure 3-B). On average, deviation from the isotropic point (absolute values) at S0A for G2 was 0.32 µm. Only the perceived neutral point in G2_F and G2_G showed little astigmatic bias. In general, the perceived neutral point remained constant across sessions for all subjects of G2.

All habitually-non corrected astigmats (G3, Fig. 3-C) showed some bias for astigmatism before astigmatic correction (S0A). The perceived neutral point was biased towards images blurred along their axis of natural astigmatism in the majority (4/7) of the cases (G3_A, G3_B, G3_E and G3_F), although in three cases (G3_C, G3_D and G3_G) the bias was orthogonal to the orientation of the natural astigmatism. Despite this difference, all subjects from G3 did show a bias towards astigmatism in the first session, which was statistically significantly different from zero (one-sample t-test; p<0.05), and shifted towards more isotropic points in later sessions. On the other hand, most subjects in G1 (except for G1_F and G1_G; one-sample t-test; p<0.04) did not show a significant shift from the isotropic point. On average, the shift for G1 (0.13 µm) was not statistically significantly different from 0, but the shift for G3 (0.34 µm) was statistically significantly different from zero (one-sample t-test; p = 0.03).

### Time-course of the adaptation effect

**Figure 4:**
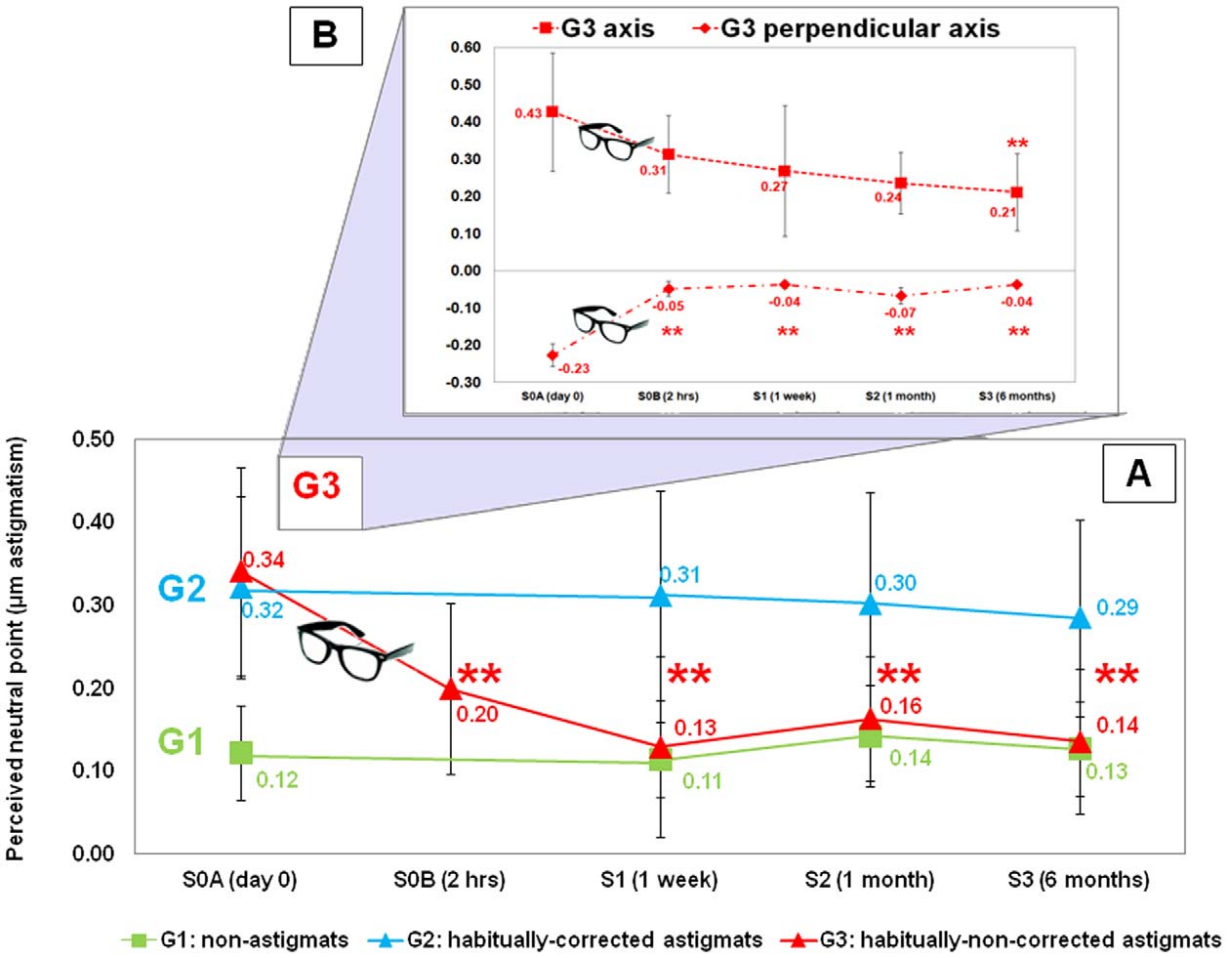
Longitudinal variations in the perception of neutral point. A. Longitudinal variation of the perceived neutral point (µm astigmatism, absolute value), averaged across subjects in each group (G1: green squares; G2: blue triangles; G3: red triangles). B. Longitudinal variations of the perceived neutral point (µm astigmatism) averaged across subjects of the 2 subgroups of G3: G3 axis (4/7 subjects) and G3 perpendicular axis (3/7 subjects). ** indicates statistically significant shifts (p<0.01), from the first session to other measurement sessions (2 hrs, 1 week, 1 month and 6 months) for G3. Error bars stand for inter-subject variability (standard deviation).

Figure 4 A shows the averaged absolute shift from isotropy as a function of session, for each group. The perceived neutral point did not change statistically across sessions for G1 and G2. However, there was a significant shift in the perceived neutral point in astigmats (G3) upon correction of astigmatism.

Very consistently, wear of the astigmatic correction shifted the perceived neutral point from the initial values. Two hours of astigmatic correction wear produced a significant shift (paired-samples t-test; t(6) = 5.494, p = 0.003) of the perceived neutral point, and a reduction of the astigmatic bias. This adaptation effect stabilized after 1 week of correction wear, and remained constant after 1 and 6 months of astigmatic correction wear, where shift was also statistically significant (one-way ANOVA F (2, 18) = 6.227, p = 0.009). On average, the perceived neutral point (absolute values) was 0.20 µm at S0B (2 hrs) and 0.14 µm at S3 (6 months).

Since perception of neutral point in G3 subjects is biased in two different ways (4 subjects biased along their axis of natural astigmatism, and 3 subjects in the perpendicular axis) in the initial session (S0A), we have also analyzed the longitudinal variations in these two subgroups independently, following the original bias towards their astigmatism (positive, G3_A, G3_B, G3_E and G3_F) or the perpendicular direction (negative, G3_C, G3_D and G3_G) (Figure 4, panel B). The shifts of the perceived neutral point at S0A were respectively 0.43 µm±0.16 and −0.23 µm±0.03 on average. Regardless the initial bias all subjects (except G3_A) shifted rapidly and consistently their perceived neutral point towards the isotropic point. Subjects with an initial bias perpendicular to the orientation of the natural astigmatism reached perceived neutral points closer to 0 (−0.04 µm±0.002 on average, at 6 months) than those with an initial bias parallel to the orientation of their natural astigmatism, which showed an average residual bias towards their natural astigmatism (0.21 µm±0.10 on average at 6 months). However, despite these differences, both sub-groups showed statistically significant longitudinal variations at 6 months (paired-samples t-test: G3 axis t (3) = 2.4999 p = 0.04; G3 perpendicular axis t (2) = 1.1999 p = 0.01).

### Adaptation and amount of natural astigmatism

**Figure 5:**
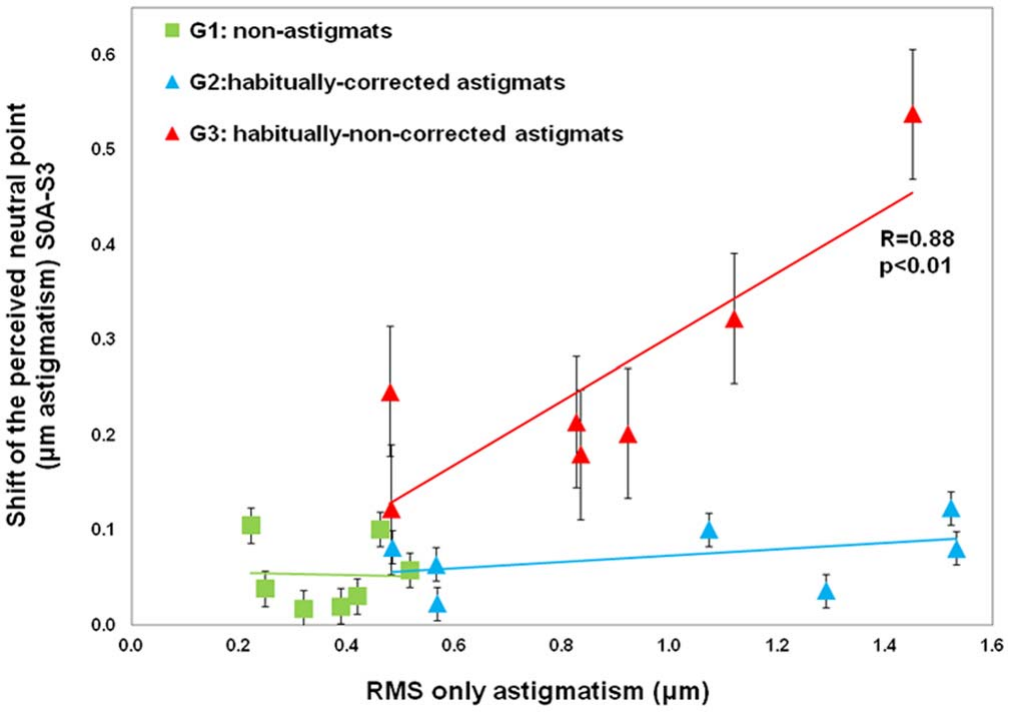
Correlation between the shift of the perceived neutral point and natural astigmatism of the subjects. Correlation between the shift of the perceived neutral point (difference between the perceived neutral point measured in the first session, S0A, and the 6-month session, S3) and the natural astigmatism for subjects. Astigmatism is represented in terms of RMS, in µm (G1: green squares; G2: blue triangles; G3: red triangles). Error bars stand for inter-subject variability (standard deviation).

The shift of the perceived neutral point was analyzed as a function of the amount of natural astigmatism (Figure 5). The astigmatism was estimated from the second order Zernike terms (RMS in microns), obtained from wavefront measurements on naked eyes, without AO correction, and averaged across sessions. The shift of the perceived neutral point was estimated for S3 (6-month) session, with respect to S0A (First session). While there was no shift in the perceived neutral point in G1 and G2, the shift of perceived neutral point was statistically correlated with the amount of natural astigmatism in G3 (p<0.01). The amount of natural astigmatism of the G3 subjects was a significant factor in the shift of the perceived neutral point across sessions (one-way ANOVA F (2, 18) = 12.936, p = 0.001).

## Discussion

Perception of blur depends on the subject's previous visual experience. Some studies have reported changes in the perceived best focus after brief exposures to sharpened or degraded images, indicating that the visual coding can very rapidly recalibrate to a changing environment. Recently, we showed that the adaptation is also selective to orientation, thus the perceived neutral point shifts after a brief exposure to images blurred by horizontal or vertical astigmatism [24]. Also, longer exposures to blur have been reported to induce changes in visual acuity [14], [16], [27].

Adaptation to astigmatism has been previously reported. However, neural adaptation of uncorrected astigmats to an astigmatic refractive correction, and the time course for this adaptation, had not been explored before. The current study shows differences in the perception of the neutral point under natural adaptation across subjects with different refractive (and corrective profiles). The measurements were conducted under full correction of both low and high order aberrations, allowing identical image quality in all subjects. The observed differences in the perception of neutral point must therefore arise from differences in the internal norm for perception of oriented blur, which is highly dependent on prior visual experience. The change of this norm after compensation of astigmatism (in a group of previously non-corrected astigmats) reveals rapid adaptability to a new astigmatic prescription.

As expected, for non-astigmats (G1) the perceived neutral point was close to isotropic, and remained stable with time (Figure 3-A, Figure 4). As per our control experiment, the effect is similar regardless the orientation of astigmatic blur (cardinal or oblique axis), indicating that the oblique effect [40] does not influence the internal code for blur orientation. This finding goes along with a previous report, which showed that oblique gratings were at least as powerful as horizontal gratings as adapting stimuli [44]. Interestingly, a previous study suggested that the oblique effect anisotropy in fact does not occur when viewing complex visual stimuli with broadband spatial content (such as natural scenes, or likely the noise stimulus used in our study [41].

All habitually-non-corrected astigmats (G3) showed a perceived neutral point shifted from isotropy before astigmatic correction wear (S0A), and in 4 out of 7 subjects, the shift occurred towards the orientation of their uncorrected astigmatism (Figure 3-C, Figure 4). The largest shifts toward the orientation of the natural astigmatism occurred in the highest astigmats (G3_B and G3_A). Unexpectedly, in three cases (G3_C, G3_D and G3_G) the shift occurred in a perpendicular orientation. This behavior might be explained by a combination of different factors: (1) a large interocular difference in the amount astigmatism (as it is the case for G3_D with an interocular difference of 4.25 D in astigmatism magnitude, see Table 1); (2) interocular difference in the astigmatic axis (as it is the case of G3_C, with a relative angle of 80° in the astigmatism axis, see Table 1); (3) a slightly hyperopic astigmatism (G3_D and G3_G, see Table 1). Interocular transfer of after-effects has been recently reported both for the amount of blur and axis of astigmatism [45]. In slightly hyperopic astigmats accommodation can shift the orientation of the blurred image. In a previous study non-corrected hyperopic astigmats showed higher performance (visual acuity) than non-astigmats in the presence of astigmatism, regardless the axis of the induced astigmatism [26]. Also, it has been shown (for large amounts of astigmatism) that meridional amblyopia is more prevalent in astigmats with both meridians myopic than in hyperopic astigmats, consistent with a more constant exposure to oriented blur in myopic than hyperopic astigmats.

Also, our study showed that wear of a newly prescribed astigmatic correction lens for a period of time shifted very systematically the perceived neutral towards isotropy, regardless of the orientation of the shift previous to the astigmatic correction. Interestingly, the shift occurred (although not in full) after two hours of lens wear, and appeared constant after one week of lens wear (and at least up to six months). Some differences in the time-scale effect between subjects of G3 were noticed. Adaptation effect was faster and almost complete after 2 hours for subjects with original bias perpendicular to their natural astigmatism, whereas subjects with original bias towards their astigmatism showed a slower and decreased adaptation effect.

The habitually corrected astigmatic group (G2), who maintained the same refractive correction throughout the study, did not show longitudinal changes in the perceived neutral point, as expected. However, interestingly, the perceived neutral point was very consistently shifted towards images blurred along the axis of their natural astigmatism (Figure 3-B, Figure 4), suggesting (unlike most subjects of G3 at the end of the study) a lack of adaptation to their astigmatic correction. We can only speculate on the reasons of this difference between G2 and G3 at the end of the study. The amount of astigmatism in the subjects of G2 was on average higher than that of G3 (G2: −2.56±1.87 D; G3: −0.39±0.64 D). In fact, G3 subjects with highest astigmatism (G3_B and G3_F), even if they experienced the largest shift in their perceived neutral point, only showed partial adaptation at the end of the study. This suggests that there may be a threshold in the amount of natural astigmatism above which adaptation may not be complete. Although we do not have evidence of clinical meridional amblyopia in the subjects of G2, numerous studies have reported orientation-specific visual performance deficits in late corrected high astigmats, which persisted despite optical correction [22], [23], [46]. Interestingly, most subjects in G2 (G2_A–F) were optically corrected after the age of 7. Another interesting element is the presence of spherical error. Most subjects of G2 had significant amounts of myopic error (corrected years back, typically simultaneously with the correction of astigmatism). The presence of defocus might have influenced the perception of blur orientation, and therefore the adaptation pattern and visual norm in those subjects. In fact G3_A (with similar amount of spherical error than the average of G2) only showed a partial adaptation. The potential impact of spherical error on the adaptation to astigmatism is consistent with differences in the perceptual responses to dioptric blur between refractive groups reported in previous studies [47], [48], [49].

Finally, long-term effects (6 months) of astigmatic correction wear in the perceived neutral point have been measured in G3 (Figure 4). Whether, if the subjects keep their correction, the adaptation to an isotropic point persists, or alternatively, a bias towards the natural astigmatism re-appears, could only be tested by monitoring the subjects of G3 after years. It has been suggested that adaptation processes can actually operate at long time-scales, show persistent after-effects, and in fact exhibit some forms of learning [1]. Vul et. al (2008) also pointed the intriguing possibility that the functional form of adaptation might change at different timescales [50]. Yehezkel et. al (2010) pointed out to the possibility of storing multiple transformations of the visual world and applying them when the need arises [7]. Alternatively to our previous hypotheses, the bias for astigmatism in the subjects of G2 might be a manifestation of one of the multiple adaptation stages in corrected astigmatic patients.

The capability for recalibration shown by subjects just given a new astigmatic prescription is of practical interest in the clinical practice. Astigmatism is routinely under-corrected on the basis that patients usually do not tolerate a full correction, and this needs to be progressively introduced [9]. An open question is whether a period of astigmatic correction wear would alter not only the perceptual bias, but also visual performance in the presence/absence of astigmatism. We had previously shown that habitually-non-corrected astigmats performed better in the presence of astigmatism, than non-astigmats with a similarly induced astigmatism [26]. If a change in visual performance is observed, it is likely the time-scale of those changes is longer than that for perceptual judgment, and involves some type of perceptual learning.

In summary, we have shown that refractive (astigmats vs. non-astigmats) and corrective (habitually-corrected or habitually-non-corrected) profiles in subjects have a large impact on their perception of oriented blur. Uncorrected astigmats appear to be naturally adapted to astigmatism, thus their perception of neutral point is shifted towards astigmatism. The observed differences in the perception of neutral point must therefore arise from differences in the internal norm for perception of oriented blur, which is highly dependent on prior visual experience. Furthermore, astigmatic correction changes significantly the perception of the neutral point in astigmatic subjects, even after a brief period of adaptation, and remains constant once stabilized.
